# Hypoperfusion of periaqueductal gray as an imaging biomarker in chronic migraine beyond diagnosis: A 3D pseudocontinuous arterial spin labeling MR imaging

**DOI:** 10.1002/brb3.70008

**Published:** 2024-09-05

**Authors:** Pan Huang, Mei Wu, Mengqi Liu, Xin Li, Yujiao Jiang, Zhiye Chen

**Affiliations:** ^1^ Department of Radiology Hainan Hospital of PLA General Hospital Sanya China; ^2^ The Second School of Clinical Medicine Southern Medical University Guangzhou China; ^3^ School of Medical Imaging Bengbu Medical College Bengbu China

**Keywords:** brain, cerebral blood flow, cerebral perfusion, chronic migraine, imaging biomarker, MRI, PCASL, periaqueductal gray matter

## Abstract

**Background:**

The periaqueductal gray (PAG) is at the center of a powerful descending antinociceptive neuronal network, and is a key node in the descending pain regulatory system of pain. However, less is known about the altered perfusion of PAG in chronic migraine (CM).

**Aim:**

To measure the perfusion of PAG matter, an important structure in pain modulation, in CM with magnetic resonance (MR) perfusion without contrast administration.

**Methods:**

Three‐dimensional pseudocontinuous arterial spin labeling (3D‐PCASL) and brain structure imaging were performed in 13 patients with CM and 15 normal subjects. The inverse deformation field generated by brain structure image segmentation was applied to the midbrain PAG template to generate individualized PAG. Then the perfusion value of the PAG area of the midbrain was extracted based on the individual PAG mask.

**Results:**

Cerebral blood flow (CBF) value of PAG in CM patients (47.98 ± 8.38 mL/100 mg min) was significantly lower than that of the control group (59.87 ± 14.24 mL/100 mg min). Receiver operating characteristic (ROC) curve analysis showed that the area under the curve was 0.77 (95% confidence interval [CI], 0.60, 0.94), and the cutoff value for the diagnosis of CM was 54.83 mL/100 mg min with a sensitivity 84.60% and a specificity 60%.

**Conclusion:**

Imaging evidence of the impaired pain conduction pathway in CM may be related with the decreased perfusion in the PAG, which could be considered as an imaging biomarker for the diagnosis and therapy evaluation.

## Graphical abstract

The cerebral blood flow (CBF) value of periaqueductal gray (PAG) in chronic migraine (CM) patients (47.98 ± 8.38 mL/100 mg min) was significantly lower than that of the control group (59.87 ± 14.24 mL/100 mg min). Red solid circle and empty circle, male CM and normal controls; Black solid circle and empty circle, female CM and normal controls.

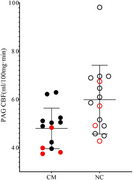

## 1 INTRODUCTION

Migraines are one of the frequent primary headache disorders and can severely affect the life quality (Tajti et al., 2023). Their pathogenesis is not clear and may be associated with genetic, environmental, and lifestyle factors (Tanaka et al., 2023). The diagnostic criteria for chronic migraine (CM) are at least 15 days per month and headache lasting longer than 3 months, with migraine lasting at least 8 days per month (Headache Classification Committee of the International Headache Society (IHS), 2018). CM affects approximately 2% of the adult population in Western countries, placing a significant burden on sufferers and their families (Lipton, 2011). The prevalence of CM in Asia ranges from 0.6% to 1.7%, affects 23 million to 65 million people in the Asia‐Pacific region, and places a great burden on individuals, society, and families (Natoli et al., 2010). Therefore, the neuromechanism of CM has recently become a focus of research (May & Schulte, 2016).

Although the pathogenesis of CM was not clearly elucidated now, the current popular etiopathogenesis of CM is cortical spreading depression (CSD) and activation of trigeminovascular system (Charles, 2018). Our previous studies had demonstrated that the amygdala (Jiang et al., 2024) and nucleus accumbens (NAc; Liu et al., 2022) presented hypoperfusion in CM while left Brodmann 38 presented hyperperfusion in episodic migraine (EM; Chen et al., 2018). Therefore, the cerebral perfusion change might be associated with migraine chronicization.

Among all the target areas of CM attacks, the periaqueductal gray (PAG) is one of the brain structures that has been focused on (Chen et al., 2016, 2017a, 2018). The PAG is located at the center of a powerful descending antinociceptive neural network (Welch et al., 2001), and is a key node in the descending pain regulatory system (DPRS; Keay & Bandler, 2002). PAG could generate multiple neurotransmitters to modulated pain state. For example, the decreased acetylcholine release in ventrolateral PAG (vlPAG) could impact the pain modulation, and the activation of cholinergic projections from the pedunculopontine tegmentum to vlPAG would relieve pain (Sullere et al., 2023). Opioids could act on the opioid receptors expressed on GABAergic neurons to inhibit gamma‐aminobutyric acid (GABA) release, which in turn would exert antinociceptive effects (Tonsfeldt et al., 2016). A previous document demonstrated that glutamatergic neurons of vlPAG projections to the rostral ventral medulla (RVM) could modulate ascending nociceptive signals and reduce the pain response (Yin et al., 2020). Therefore, the structural and functional study of PAG would appear more important in chronic pain.

A previous study on the morphometry of the PAG showed increased density of the PAG in patients with CM (Rocca et al., 2006), and texture analysis suggested that the contrast of texture feature parameters of structural images was increased in patients with CM (Chen et al., 2018). Further volumetric analysis revealed that the cerebellum and brainstem were smaller in CM patients compared to healthy controls (Bilgiç et al., 2016), while the volume of the PAG did not show significant changes in CM compared with normal controls (NCs) and EM (Chen et al., 2017b). Although all the above studies on structural and texture features noted the subtle changes of PAG in CM, functional observation appeared increasingly important to further explore the neuromechanism of CM.

Three‐dimensional pseudocontinuous arterial spin labeling (3D‐PCASL; Li et al., 2023) is widely used in clinical practice, and the previous study had demonstrated the regional brain regions with increased perfusion in EM (Chen et al., 2018) and hypoperfusion in the NAc in CM (Liu et al., 2022). However, the perfusion state of the PAG has not been previously investigated.

In this study, we hypothesize that CM patients may present altered perfusion in PAG. To address this hypothesis, we prospectively obtained the 3D‐PCASL images and brain structural images from 13 CM patients and 15 normal volunteers. The cerebral blood flow (CBF) and volume of PAG were automatically extracted and compared to detect the perfusion change of PAG to elucidate the neuromechanism of PAG dysfunction in CM pathogenesis.

## 2 MATERIALS AND METHODS

### 2.1 Subjects

Thirteen CM patients (four male/nine female) recruited from the Headache Clinic, Chinese PLA General Hospital from 2013 to 2015, and 15 normal volunteers (four male/11 female). The inclusion criteria for CM patients should meet the following criteria: (1) Meeting the international headache diagnostic criteria (III β‐version): Migraine 1.1, 1.2, and 1.3; (2) no preventive medication for migraine in the past 3 months; (3) all patients were right‐handed. The exclusion criteria were listed as follows: (1) history of chronic hypertension, diabetes, coronary artery disease, cerebrovascular disease, and so forth; (2) history of long‐term alcohol abuse, smoking, and use of other drugs; (3) history of brain trauma; (4) history of neuropsychological disorders. Normal subjects were recruited from the society and were required to meet the following conditions: (1) no history of headache; (2) right‐handedness. The exclusion criteria were the same as for CM. A visual analog scale (VAS) was used to assess headache severity at CM. All subjects were assessed with the Hamilton Anxiety Scale (HAMA; Maier et al., 1988), the Hamilton Depression Scale (HAMD), and the Mini‐Mental State Examination (MMSE; Hamilton, 1967). All the patients underwent magnetic resonance imaging (MRI) scanning at least days after last migraine attack. Written informed consents were obtained from all participants according to the approval of the ethics committee of the Chinese PLA General Hospital.

### 2.2 Magnetic resonance imaging

Images were acquired on a GE 3.0T MRI scanner (DISCOVERY MR750, GE Healthcare) with a conventional eight‐channel quadrature head coil. Patients were in the supine position, and a sponge pad was placed on both sides of the head to prevent head movement. The imaging sequence and parameters were set as follows: (1) Brain structure data were obtained using an axial 3D T1‐weighted fast‐spoiled gradient‐recalled echo (3D T1‐FSPGR) sequence with a slice number = 360, repetition time = 6.3 ms, echo time = 2.8 ms, inversion time = 450 ms, flip angle = 15°, field of view = 25.6 cm × 25.6 cm, matrix = 256 × 256, acquisition times = 1, slice thickness = 1 mm; (2) 3D PCASL was used for brain perfusion imaging. Imaging parameters were set as follows: repetition time = 5128 ms, echo time = 15.9 ms, flip angle = 111°, field of view = 20 cm × 20 cm, matrix = 1024 × 8 (spiral acquisition), slice thickness = 3.0 mm, postlabeling delay time (PLD) = 1.5 s. CBF images could be automatically obtained after 3D PCASL scan. (3) Traditional oblique transverse T2‐weighted imaging and diffusion‐weighted imaging were scanned to exclude the subjects with obvious brain lesions.

### 2.3 Image processing

**FIGURE 1:**
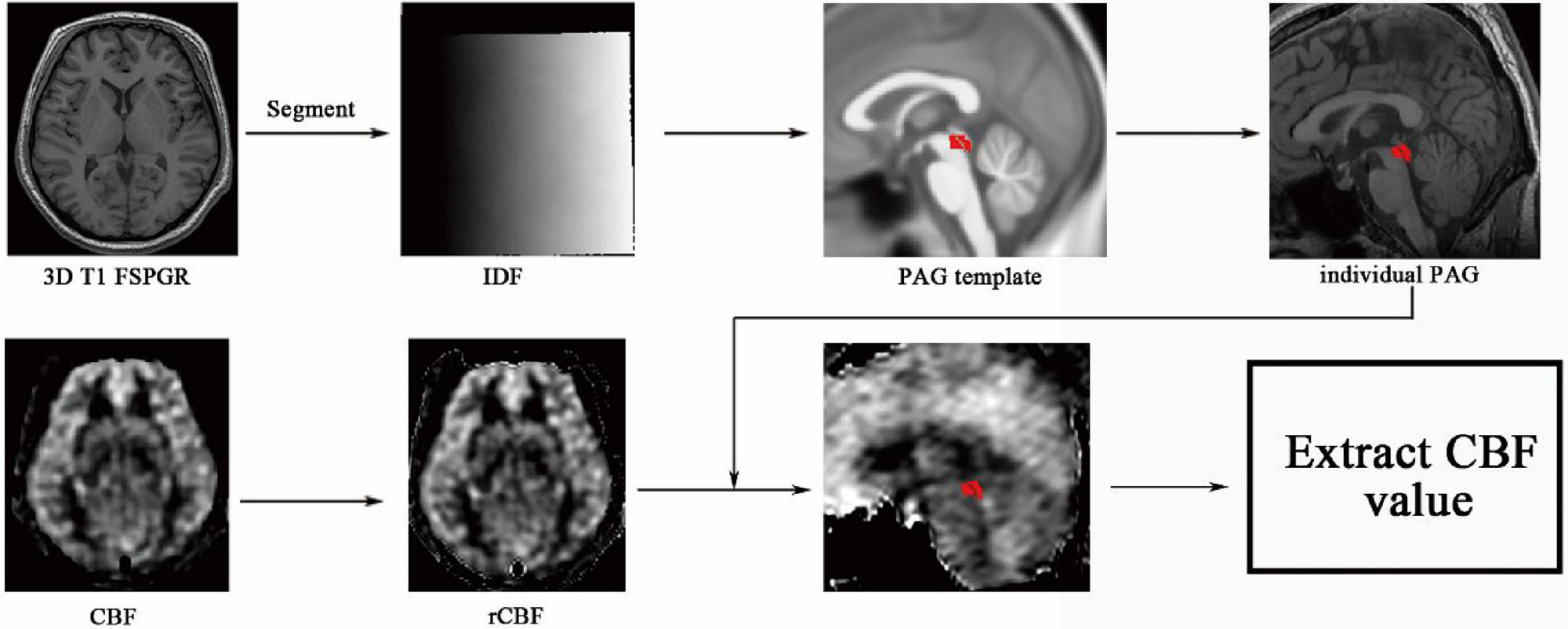
The flowchart for the cerebral blood flow (CBF) extraction of the periaqueductal gray (PAG) matter. IDF, inverse deformation field; 3D T1‐FSPGR, three‐dimensional T1‐weighted fast‐spoiled gradient‐recalled echo.

Image analysis was performed using Statistical Parametric Mapping 12 (SPM 12) and the CAT12 plugin (http://www.fl.ion.ucl.ac.uk/spm/). The running environment was MATLAB 7.6 (The Mathworks). The image processing steps are as follows (Figure 1): (1) The brain structure image (3D T1‐FSPGR) is segmented to generate an inverse deformation field (IDF); (2) The PAG template was created based on the mni_icbm152_gm_tal_nlin_asym_09a template using MRIcron software (http://www.mricro.com); (3) The subject's IDF is applied to the PAG template to generate an individual PAG mask (wPAG); (4) Each individual PAG mask was checked and confirmed using MRIcron software; (5) The volume of each individual PAG was extracted; (6) The CBF images were coregistered with the brain structure images, and then a realigned CBF was generated; (7) The individual PAG mask was overlaid on the realigned CBF images, and then the CBF value of the PAG was extracted.

### 2.4 Statistical analysis

Quantitative data with a normal distribution are presented in the form expressed by mean ± SD, and non‐normally distributed quantitative data expressed by median (minimum, maximum). Chi‐square test was used for comparison of sexes; the independent‐sample *t*‐test was used for comparison of age, HAMA, HAMD, and PAG volume between groups; Mann–Whitney *U*‐test was used for comparison of MMSE scores between groups. The generalized linear model was used to compare CBF between groups with age and sex as covariates. Receiver operating characteristic (ROC) curve analysis was applied for the diagnostic efficacy of PAG perfusion to discriminate CM from NC, and the area under the curve (AUC) was accepted as an appropriate diagnostic valuable with the AUC set at >0.7 (Ueno et al., 2021). Correlation between quantitative data was analyzed by partial correlation analysis with age and sex as control variables. A *p* value of <.05 was set as a significant difference for statistical analysis.

## 3 RESULTS

There was no significant difference between the two groups in terms of age (*T* value = 0.78, *p* = .44) and sex (*χ* ^2^ = 0.03, *p* = .86; Table 1). The mean disease duration of CM was 8 (3, 30) years, and the VAS score of pain severity was 5 ± 1.56. The HAMA score of CM was significantly higher than that of the control group (*T* value = 3.69, *p* = .001). There was no significant difference in HAMD and MMSE scores between the two groups.

**TABLE 1:** Comparisons of the clinical characteristic between CM and NC.

	CM	NC	*T* value	*p* value
Numbers (F/M)	13 (9/4)	15 (11/4)	0.03^a^	.86
Age (years)	40.69 ± 8.60	38.00 ± 9.00	0.78	.44
DD	8 (3, 30)^c^	NA	NA	NA
VAS	5 ± 1.56	NA	NA	NA
HAMA	21.38 ± 11.7	9.73 ± 3.39	3.69	.001
HAMD	16.46 ± 10.58	15.73 ± 2.91	0.26	.80
MMSE	27.00 ± 3.19	28.00 (26.00, 30.00)^c^	74.50^b^	.28

Abbreviations: CM, chronic migraine; DD, disease duration; HAMA, Hamilton Anxiety Scale; HAMD, Hamilton Depression Scale; MMSE, Mini‐Mental State Examination; NA, not available; NC, normal control; VAS, visual analog scale.

^a^ Chi‐square test.

^b^ Mann–Whitney *U*‐test.

^c^ Median (Minimum, Maximum).

### 3.1 Comparison of the perfusion and volume of PAG between CM and NC

**FIGURE 2:**
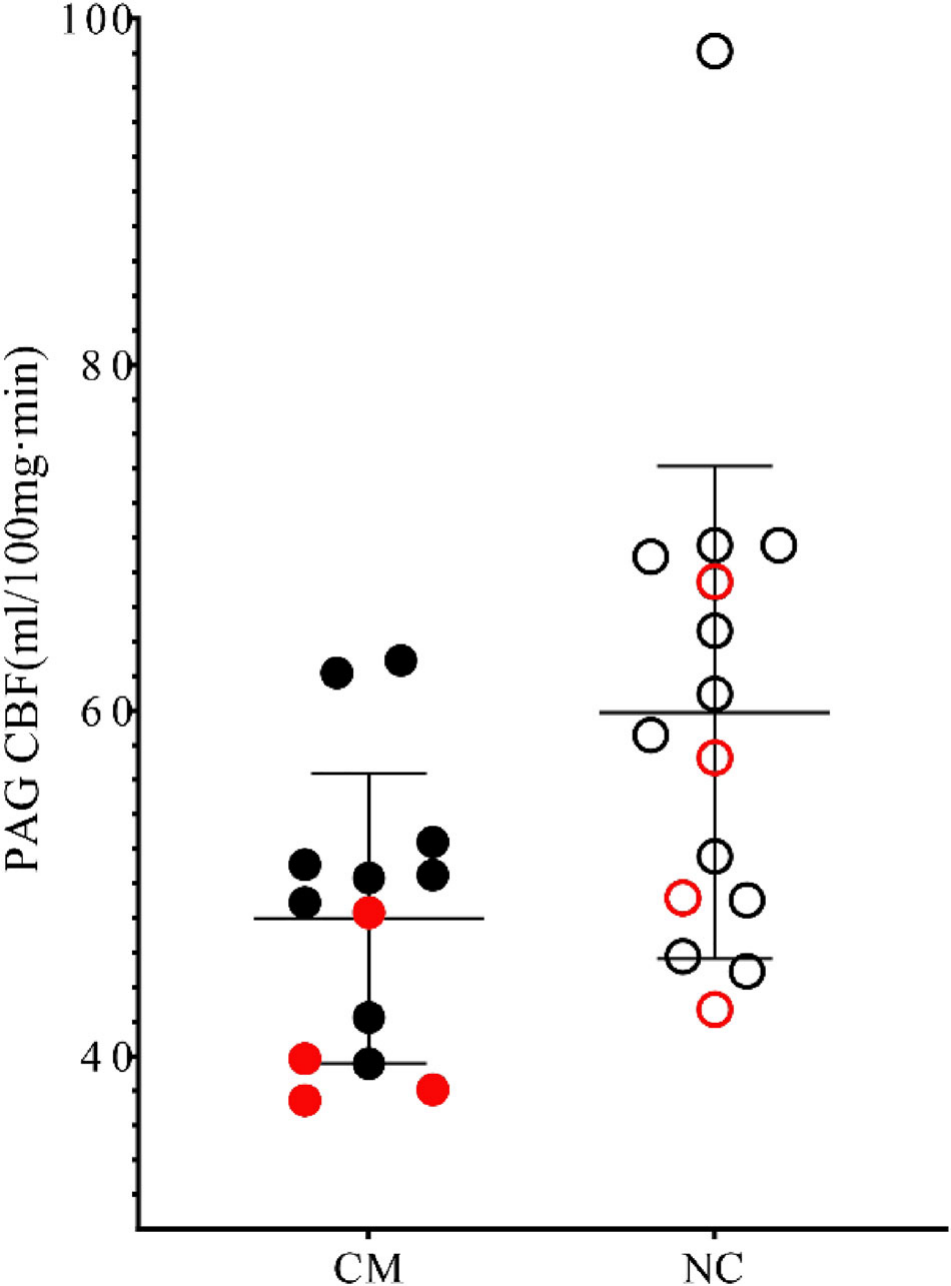
The cerebral blood flow (CBF) value of periaqueductal gray (PAG) matter in chronic migraine (CM) and normal control (NC). Red solid circle and empty circle, male CM and NCs; Black solid circle and empty circle, female CM and NCs.

Perfusion of PAG in the CM group (47.98 ± 8.38 mL/100 mg min) was significantly lower than that in the control group (59.87 ± 14.24 mL/100 mg min; *F* = 6.58, *p* = .02; Figure 2). For PAG volume, there was no significant difference between CM (0.45 ± 0.06 mL) and NC (0.43 ± 0.05 mL; *F* = 0.79, *p* = .38).

### 3.2 ROC analysis of the CBF value of the PAG in CM

**FIGURE 3:**
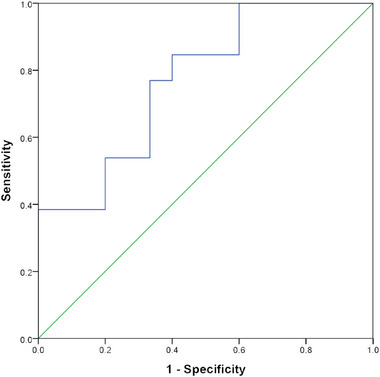
Receiver operating characteristic (ROC) of periaqueductal gray matter perfusion for the diagnosis of chronic migraine (CM) from normal control (NC). The blue line, ROC curve; the green line, reference line.

ROC curve analysis showed that the AUC was 0.77 (0.60, 0.94; 95% confidence interval [CI]), the cutoff value was 54.83 mL/100 mg min with sensitivity 84.60% and specificity 60% (Figure 3).

### 3.3 Correlation analysis of the CBF value of the PAG with the clinical variables in CM

There was no significant correlation between CBF value in PAG and VAS score, HAMA score, PAG volume, and disease progression in CM patients (*p* > .05).

## 4 DISCUSSION

3D PCASL can reflect the microvascular perfusion of the brain, and it is not necessary to inject contrast agent and is less affected by magnetic artifacts. The appearance of CM may be closely related to the changes in CBF (Maniyar & Goadsby, 2013). In this study, 3D PCASL was used to investigate the perfusion state of the PAG in patients with CM to understand the pathophysiological changes of CM. The current study showed that hypoperfusion in the PAG was identified in CM, suggesting that microvascular perfusion injury may play a key role in the progression of CM attacks.

It is well known that the brain regulates pain perception through the DPRS, and the PAG is only the key node of the DPRS (Wu et al., 2014). The PAG is located in the midbrain and is an anatomical and functional interface between the forebrain and lower brainstem (Benarroch, 2012), which plays a central role in mediating pain (Depaulis et al., 1994) and receives prefrontal cortical projections (An et al., 1998). The PAG also plays a dual role in controlling (including inhibiting and promoting) of nociceptive information transmission in the spinal dorsal horn and trigeminal nucleus (Benarroch, 2012). Therefore, the PAG is an important receptor for the response to pain, which may be related to the fact that the PAG contains many types of neurotransmitters (such as l‐glutamate, γ‐aminobutyric acid, opioids, etc.; Benarroch, 2012).

The PAG is a key component of the network associated with pain regulation and nociceptive input (Keay & Bandler, 2002; Lumb, 2004), which involves the ascending and descending pain regulation system, and the chronic pain is commonly associated with the descending pain modulation (Hemington & Coulombe, 2015). It is known that PAG receives inputs from cortical sites and has reciprocal connections with the amygdala (Helmstetter et al., 1998), rostral anterior cingulate cortex (Petrovic et al., 2002) and rostroventromedial medulla (Ossipov et al., 2014). More recent studies demonstrated that decreased descending inhibition might be an important factor in pain chronification (Ossipov et al., 2014). This study indirectly confirmed that the hypoperfusion of PAG might influence the pain modulation of PAG region. Our previous study also demonstrated that the decreased functional connectivity of PAG with right amygdala presented in CM compared with NCs (Chen et al., 2017), which might be associated with the hypoperfusion of PAG. Therefore, it could be speculated that the hypoperfusion of PAG might be associated with the decreased pain modulation network activity of PAG.

Neurovascular coupling (NVC) has been applied to assess abnormal brain metabolism in migraine, which was also one of features of the “migraine state” (Fabjan et al., 2015). The hypoperfusion would impair the NVC of PAG in CM, that is, the close spatial and functional relationship between PAG and microvessel wall cells (pericytes, smooth muscle, and endothelial cells; Stanimirovic & Friedman, 2012). The interrupted migraine state would decreased the cerebral flood flow, and then impact the local oxygen and lactate concentration, and then damage the response of microvessels (Gordon et al., 2008). Therefore, PAG microvascular damage may be closely related to the occurrence of CM, and may be worth further investigation, and MR spectroscopy revealed significantly higher glutamate/glutamine (Glx) levels in the PAG in CM compared with NC (Wang et al., 2022). Interestingly, the current study showed that microvascular perfusion of the PAG was decreased in CM, and further correlation analysis revealed that there was no significant correlation with PAG volume in CM. Therefore, modern neuroimaging techniques can observe the neurofunctional changes in patients with CM to further explore the neuromechanism of migraine attacks (Chen et al., 2019, 2021). The functional state of neurons (activation or deactivation) is related to the changes in the local CBF. Therefore, the altered CBF could be used as a reliable index to evaluate the functional state of neurons (Bilgiç et al., 2016). The decreased perfusion of the PAG might reflect the dysfunction of local neurons in the PAG in the current study. Therefore, microvascular perfusion imaging may provide a new way to evaluate the neuronal functional status of migraine patients. In this study, ROC analysis provided a cutoff value (54.83 mL/100 mg min) for perfusion of the PAG to diagnose CM with a moderate degree of diagnostic efficacy. Therefore, quantitative perfusion measurement in the PAG could be useful for clinical staging of migraine patients. Calcitonin gene‐related peptide (CGRP) a vasodilator neuropeptide that plays a crucial role in the pathomechanism of migraine and pain transmission in the somatosensory nervous system (Tajti et al., 2023). The innovated therapeutic strategy is based on human and fully humanized monoclonal antibodies (mAbs) targeting CGRP and CGRP receptors, which had revealed the high efficacy and safety of these pharmacons in CM patients (Edvinsson et al., 2018). The measurement of PAG perfusion state could efficiently provide a relatively easy and convenient evaluating tool for the treatment planning and monitoring of the therapeutic efficacy. Future studies should focus on the clinical investigation of how to predict whether migraine patients will gradually progress from episodic to CM, in which the altered microvascular perfusion of the PAG may play an important role.

Migraine without aura has been frequently observed in patients with spontaneous dissection of the internal carotid artery, which is a common cause of ischemic stroke in young people (Lyden, 2017; Sharif et al., 2010). The significant decrease of perfusion in the PAG confirmed in this study could alter the response of the neurovascular unit and then trigger a migraine attack (Lyden, 2017). Further longitudinal observations should be conducted in the future to determine the causal relationship between migraine and changes in the brain microvascular system. It has been demonstrated that CM may exhibit changes in CBF velocities (Lee et al., 2017), and our study further identified the decreased perfusion of the PAG in CM from the perspective of local perfusion. It is reasonable to believe that more and more new technologies could be used to study the CBF changes in patients with CM and explore their neuromechanism.

Although a voxel‐based morphometry study found that the volume of the PAG increased significantly in patients with CM based on voxel level (Yu et al., 2021). However, PAG volume showed no significant difference compared with the NC in the current study, which was consistent with our previous study (Chen et al., 2017b). Further correlation analysis showed that there was no significant correlation between CBF value and PAG volume. Therefore, it was reasonable to speculate that the altered PAG perfusion might occur earlier than the changes in PAG volume, and the evaluation of the PAG perfusion would improve the understanding of the neuromechanism of CM.

Limitations of this study include: (1) Although the current study identified PAG hypoperfusion in CM as a preliminary study, the small sample size may increase the systematic error of the study. (2) The perfusion imaging method used in this study is noncontrast perfusion imaging, and permeability imaging or dynamic susceptibility contrast perfusion imaging may be used in future studies to generate more perfusion parameters. (3) According to the previous studies (Binnewijzend et al., 2013; Chappell et al., 2021), partial volume effect (PVE) may underestimate gray matter CBF. In the present study, all CBF images were automatically acquired using MR scanner, and PVE was not performed. (4) The enrolled patients came from all over China and were far from our hospital, therefore, which resulted in poor compliance for the longitudinal study. In the future, we would try to perform the longitudinal study by enrolling the local CM patients to provide a dynamic process of altered PAG perfusion and provide deeper insights into the pathophysiology of CM.

## 5 CONCLUSION

In conclusion, this study found that decreased perfusion in the PAG may be direct imaging evidence of pain pathway damage in CM, and perfusion measurement of the PAG can be used as an imaging biomarker for the evaluation of diagnosis and therapy.

## AUTHOR CONTRIBUTIONS

**Pan Huang**: Writing—original draft; data curation; investigation; methodology. **Mei Wu**: Methodology; investigation. **Mengqi Liu**: Methodology; investigation. **Xin Li**: Methodology; investigation. **Yujiao Jiang**: Methodology; investigation. **Zhiye Chen**: Conceptualization; writing—review and editing; methodology; software.

## CONFLICT OF INTEREST STATEMENT

The authors declare no conflicts of interest.

## FUNDING INFORMATION

This study did not receive any funding support.

### PEER REVIEW

The peer review history for this article is available at https://publons.com/publon/10.1002/brb3.70008.

## Data Availability

Data sharing is not applicable to this article as no new data were created or analyzed in this study.
